# Association Between Body Mass Index and Treatment Toxicity in Patients with Metastatic Renal Cell Carcinoma Treated with Sunitinib or Pazopanib

**DOI:** 10.3390/cancers18172730

**Published:** 2026-08-23

**Authors:** Ahmet Kürşad Dişli, Ayşe Nuransoy Cengiz, Muhammet Cengiz, İrfan Buğday, Sinan Yıldırım, Rıdvan Gönül, Oktay Bozkurt, Mevlüde İnanç, Metin Özkan

**Affiliations:** 1 Department of Medical Oncology, Kırşehir Education and Research Hospital, Kırşehir 40100, Turkey; 2 Department of Medical Oncology, Erciyes University, Kayseri 38039, Turkey; 3 Department of Medical Oncology, Kayseri City Hospital, Kayseri 38080, Turkey

**Keywords:** sunitinib, pazopanib, toxicity, body mass index, renal cell carcinoma

## Abstract

**Background/Objectives:** Sunitinib and pazopanib are administered at fixed doses in patients with metastatic renal cell carcinoma (mRCC), without adjustment for body composition. This study aimed to investigate the association between body mass index (BMI) and treatment-related toxicity and treatment discontinuation in patients with mRCC receiving first-line sunitinib or pazopanib. **Methods:** A total of 136 patients with mRCC treated with sunitinib (*n* = 90) or pazopanib (*n* = 46) were retrospectively evaluated. BMI was compared according to toxicity grade (1–2 and 3–4) and treatment discontinuation using Student’s *t*-tests and chi-square/Fisher’s exact tests. Given the limited number of grade 3–4 toxicity events, Firth’s penalized logistic regression was used for multivariable analysis, with adjustment for age, sex, and treatment type. BMI was evaluated as a continuous variable in the primary model and categorically in a supportive analysis. *p* < 0.05 was considered statistically significant. **Results:** Higher BMI was significantly associated with grade 1–2 (*p* < 0.001) and grade 3–4 toxicity (*p* < 0.001). In the Firth penalized multivariable analysis, higher BMI remained independently associated with grade 3–4 toxicity (OR per 1 kg/m^2^ increase, 1.160; 95% CI, 1.057–1.283; *p* = 0.0015). Increasing age (OR per 1-year increase, 1.061; 95% CI, 1.021–1.106; *p* = 0.0022) and sunitinib treatment (OR, 2.629; 95% CI, 1.104–6.785; *p* = 0.028) were also independently associated with grade 3–4 toxicity. In the supportive categorical analysis, obesity (BMI ≥ 30 kg/m^2^) was associated with higher odds of grade 3–4 toxicity compared with BMI < 25 kg/m^2^ (OR, 4.173; 95% CI, 1.533–11.982; *p* = 0.005). **Conclusions:** A higher BMI was associated with a greater likelihood of grade 3–4 TKI-related toxicity in patients with mRCC receiving fixed-dose sunitinib or pazopanib. BMI may represent a practical clinical marker for identifying patients who warrant closer toxicity monitoring.

## Simple Summary

Renal cell carcinoma that has spread to other parts of the body is often treated with oral drugs called sunitinib or pazopanib, which are given at the same fixed dose to every patient regardless of body size. This study examined whether a patient’s body mass index (BMI) affects how well these drugs are tolerated. Medical records of 136 patients with metastatic kidney cancer treated with sunitinib or pazopanib were reviewed, and side effects and treatment discontinuation were compared according to BMI. Patients with a higher BMI, especially those classified as obese, experienced more severe side effects. These findings suggest that BMI could help identify patients who need closer monitoring during treatment, although further prospective studies are needed to confirm these findings.

## 1. Introduction

Renal cell carcinoma (RCC) is most commonly diagnosed between 60 and 70 years of age and occurs more frequently in men than in women [1]. The disease is frequently asymptomatic in early stages, and ~25% of patients present with metastatic disease at the time of diagnosis. Clear cell carcinoma (ccRCC) is the most common subtype of RCC, accounting for ~65–75% of all cases [2]. RCC comprises a heterogeneous group of malignancies with distinct histopathological and molecular characteristics. The 2022 World Health Organization classification recognizes several major categories of renal tumors and has expanded the classification to include molecularly defined renal carcinomas, reflecting the increasing importance of molecular alterations in RCC diagnosis and classification [3].

In ~75–80% of ccRCC cases, a disruption of heterozygosity at the Von Hippel–Lindau (VHL) tumor suppressor gene, located on chromosome 3p25, is present, resulting in overproduction of vascular endothelial growth factor (VEGF). VEGF is a key factor involved in tumor angiogenesis and plays a central role in the development and progression of several cancers, including RCC. Several agents targeting the VEGF signaling pathway have been approved for the treatment of metastatic RCC (mRCC). These agents inhibit VEGF signaling, resulting in hypoxic stress and tumor cell apoptosis, ultimately leading to vascular endothelial cell apoptosis [4,5,6].

The systemic treatment landscape of advanced RCC has evolved substantially with the introduction of immune checkpoint inhibitors (ICIs) and ICI-based combination therapies. Contemporary first-line treatment strategies for advanced clear-cell RCC include dual immune checkpoint inhibition and combinations of ICIs with VEGF receptor-targeted TKIs, which have demonstrated improved clinical outcomes compared with sunitinib in randomized phase III trials [7,8]. Nevertheless, VEGF-targeted TKIs, including sunitinib and pazopanib, remain clinically relevant treatment options in selected patients.

Sunitinib and pazopanib are orally administered drugs that inhibit tyrosine kinases associated with VEGF, platelet-derived growth factor receptors, and KIT receptors. Previous prospective studies have reported that both agents can significantly improve progression-free survival (PFS) and achieve comparable overall survival outcomes in patients with mRCC [9,10,11].

Both sunitinib and pazopanib are administered at fixed doses regardless of patient-specific anthropometric variables, including BMI. However, this fixed-dose approach does not account for interindividual variability in drug metabolism, distribution, and toxicity profiles. Previous studies have suggested that body composition, including skeletal muscle mass and visceral adiposity, may be associated with sunitinib-related toxicity; however, the available evidence remains limited and findings across different body composition measures are not entirely consistent [12,13]. The association between BMI and treatment-related toxicity remains unclear and is not routinely considered when choosing treatment doses. Clarifying this association may help identify patients who require closer toxicity monitoring during treatment.

The present study aimed to investigate the association between BMI and treatment-related toxicity and treatment discontinuation in patients with mRCC receiving first-line therapy with sunitinib or pazopanib.

## 2. Materials and Methods

### 2.1. Study Design and Patients

This retrospective cohort study was conducted at the Department of Medical Oncology, Erciyes University. Only patients with histologically confirmed mRCC were included in the study; patients treated for other malignancies were not eligible. A consecutive sampling approach was used, whereby all patients who received first-line sunitinib or pazopanib during the study period and met the predefined eligibility criteria were included. A total of 136 patients diagnosed with mRCC who received first-line treatment with either sunitinib or pazopanib were included. Eligible patients were ≥18 years of age, with histologically confirmed mRCC, and treated with either sunitinib or pazopanib as first-line therapy. Patients with incomplete medical records or who had not undergone follow-up evaluations were excluded (n = 5). The final cohort consisted of 136 patients, including 94 males (69.1%) and 42 females (30.9%), with a median age of 59 years (range, 28–92 years).

**Figure 1:**
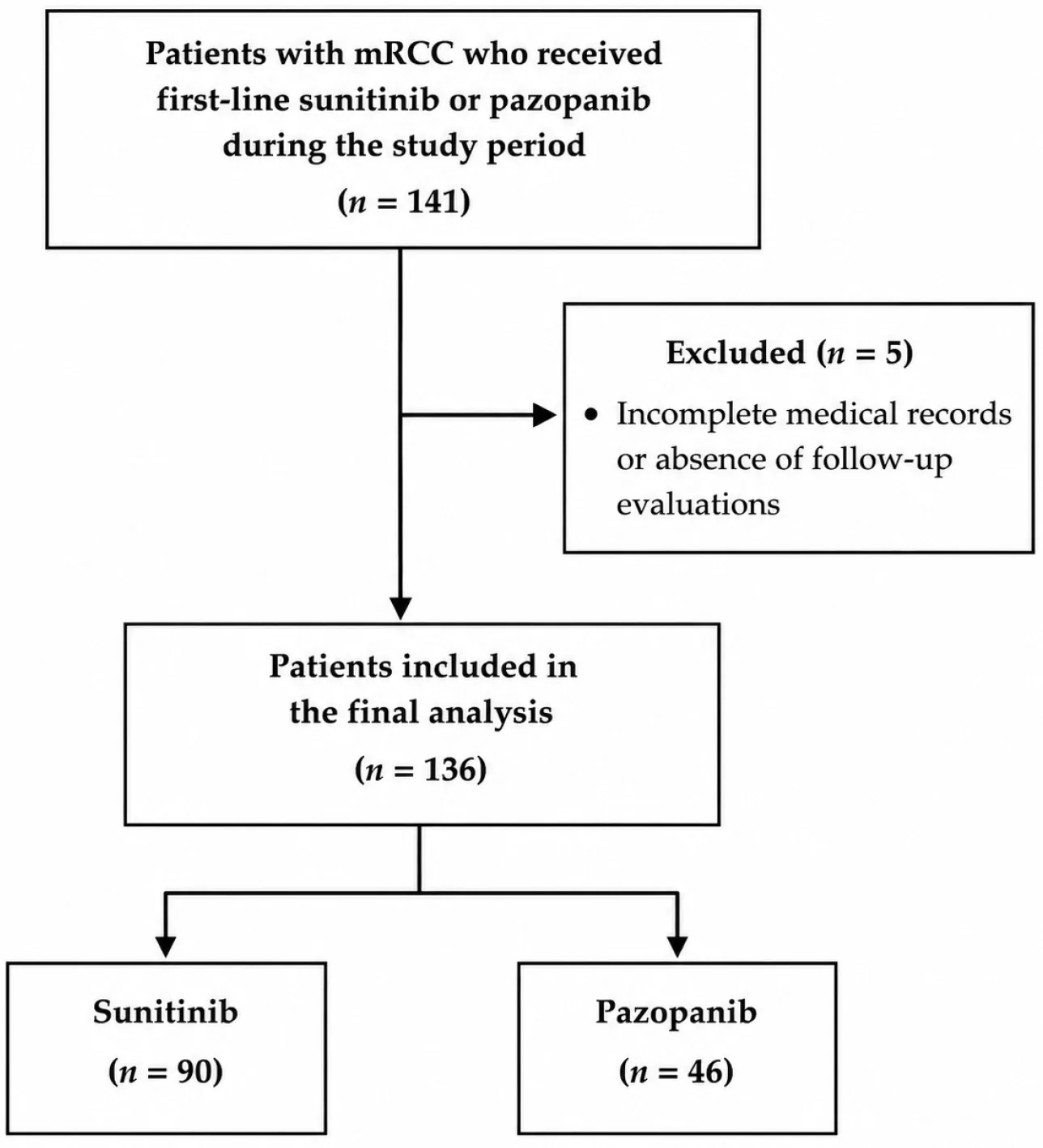
Flow diagram of patient selection and study cohort.

Among the included patients, first-line sunitinib or pazopanib treatment was initiated between March 2013 and May 2024. Clinical data were retrospectively collected through an administrative data cutoff of October 28, 2024. The patient selection process is shown in Figure 1.

### 2.2. Study Variables

Demographic, clinicopathological, and treatment-related data were retrospectively collected from electronic medical records. Variables collected included age, sex, BMI, body surface area (BSA), nephrectomy status, histological subtype, International Metastatic RCC Database Consortium (IMDC) risk score, Eastern Cooperative Oncology Group (ECOG) performance status, treatment-related toxicities graded according to National Cancer Institute Common Terminology Criteria for Adverse Events (CTCAE) version 5.0, dose reduction, and treatment discontinuation. Baseline BMI was calculated as weight (kg)/height^2^ (m^2^), and patients were categorized according to the World Health Organization (WHO) classification as underweight (BMI < 18.5 kg/m^2^), normal weight (BMI, 18.5–24.9 kg/m^2^), overweight (BMI, 25.0–29.9 kg/m^2^), and obese (BMI, ≥30.0 kg/m^2^). Because only four patients were underweight, underweight and normal-weight patients were combined into a BMI < 25.0 kg/m^2^ group for categorical comparative analyses. Accordingly, categorical analyses were performed using BMI < 25.0, 25.0–29.9, and ≥30.0 kg/m^2^, while BMI was retained as a continuous variable in the primary multivariable analysis.

Grade 1–2 and grade 3–4 toxicity variables indicated whether a patient experienced at least one treatment-related toxicity within the respective grade range during treatment and were not mutually exclusive. Thus, patients who developed grade 3–4 toxicity could also have experienced grade 1–2 toxicity during treatment. Grade 3–4 treatment-related toxicity was defined as the primary toxicity outcome. Because dose reduction was completely concordant with grade 3–4 toxicity in this cohort, it was considered a clinical consequence of severe toxicity rather than a separate analytical endpoint. Treatment discontinuation due to toxicity was evaluated separately. Specific adverse-event types were not systematically captured as separate variables in the study database and therefore could not be reliably evaluated retrospectively.

### 2.3. Treatment Plan

Sunitinib was administered at the standard starting dose of 50 mg/day using a 4 weeks-on/2 weeks-off schedule. Pazopanib was administered at 800 mg/day on a continuous daily schedule. All patients received the standard starting doses, and none received an initial dose reduction. The severity of treatment-related toxicity was retrospectively evaluated by the treating physician according to CTCAE version 5.0 at each clinical visit and documented in the electronic medical database.

Treatment duration was defined as the interval between initiation of first-line therapy and documented disease progression or treatment discontinuation. Complete treatment-duration data were available for 97 patients (71.3%), and missing treatment-end dates were not imputed. Because the exact date of first treatment-related toxicity was not systematically recorded, a formal time-to-toxicity analysis could not be performed.

### 2.4. Statistical Analysis

Descriptive and univariable statistical analyses were performed using IBM SPSS Statistics for Windows, version 25.0 (IBM Corp., Armonk, NY, USA). Descriptive statistics are expressed as the mean ± standard deviation, medians (range), and percentages, as appropriate. Data normality was assessed using the Kolmogorov–Smirnov test. For continuous variables, comparisons between two groups were performed using an independent-samples *t*-test, whereas categorical variables were analyzed using Pearson’s chi-square test or Fisher’s exact test, as appropriate. *p* < 0.05 was considered indicative of a statistically significant difference. Given the limited number of grade 3–4 toxicity events, Firth’s penalized logistic regression was used for the primary multivariable analysis to reduce small-sample and sparse-data bias. BMI was evaluated as a continuous variable in the primary model, with adjustment for age as a continuous variable, and sex and treatment type (sunitinib vs. pazopanib) selected as clinically relevant potential confounders. Adjusted odds ratios (ORs) and 95% profile penalized-likelihood confidence intervals (CIs) were reported. A supportive categorical analysis evaluated BMI < 25.0, 25.0–29.9, and ≥30.0 kg/m^2^, using BMI < 25.0 kg/m^2^ as the reference category. A conventional multivariable logistic regression analysis is additionally presented in Supplementary Table S1, and an exploratory BSA analysis is provided in Supplementary Table S2.

## 3. Results

### 3.1. Patient Characteristics

A total of 136 patients with mRCC were included, consisting of 94 males (69.1%) and 42 females (30.9%). The mean BMI was 26.28 ± 4.50 kg/m^2^. The sociodemographic and clinical characteristics of the patients are summarized in Table 1. Treatment duration data were available for 97 patients (71.3%). At the time of data cutoff, 26 patients remained on treatment and were censored, while one patient had an undocumented treatment discontinuation date. The overall median treatment duration was 9.2 months (sunitinib group, 9.0 months, range 1.1–75.7 months, n = 62; pazopanib group, 12.6 months, range 1.8–51.2 months, n = 35). As treatment duration was defined as the interval between the start of first-line therapy and disease progression or drug discontinuation, it reflects time to treatment discontinuation (TTD) rather than progression-free survival.

### 3.2. Treatment Related Toxicity, Dose Reduction, and Treatment Discontinuation

Grade 3–4 toxicity and dose reduction were perfectly concordant in this cohort. All 41 patients (30.1%) who experienced grade 3–4 adverse events required dose reduction, whereas no patient underwent dose reduction in the absence of grade 3–4 treatment-related toxicity. Dose reduction was therefore considered a clinical consequence of severe toxicity rather than a separate analytical endpoint. All 12 patients who discontinued treatment had previously developed grade 3–4 toxicity; however, 29 of the 41 patients with grade 3–4 toxicity were successfully managed with dose reduction and were able to continue therapy. Of the 41 patients who developed grade 3–4 toxicity, all had also experienced at least one grade 1–2 toxicity during treatment; therefore, these toxicity categories were not mutually exclusive.

### 3.3. BMI Is Associated with Treatment-Related Toxicity

As shown in Table 2, among patients treated with sunitinib, BMI values were significantly higher in those who developed grade 1–2 toxicity (*p* < 0.001) and grade 3–4 toxicity (*p* = 0.008). In patients treated with pazopanib, BMI was significantly higher among those who developed grade 3–4 toxicity (*p* = 0.005), whereas no significant association was observed for grade 1–2 toxicity (*p* = 0.141). BMI was not significantly associated with treatment discontinuation in either treatment group.

Categorical BMI analyses are presented in Table 3. In the total cohort, grade 3–4 toxicity occurred in 14 of 59 patients (23.7%) with BMI < 25.0 kg/m^2^, 11 of 49 (22.4%) with BMI 25.0–29.9 kg/m^2^, and 16 of 28 (57.1%) with BMI ≥ 30.0 kg/m^2^ (*p* = 0.002). In the pazopanib subgroup, grade 3–4 toxicity was also more frequent in patients with BMI ≥ 30.0 kg/m^2^ (*p* = 0.001). No significant association between BMI category and treatment discontinuation was observed in the total cohort or within either treatment group.

### 3.4. Multivariable Analysis

In the Firth penalized multivariable logistic regression model, higher BMI remained independently associated with grade 3–4 treatment-related toxicity after adjustment for age, sex, and treatment type. Each 1 kg/m^2^ increase in BMI was associated with a 16.0% increase in the odds of grade 3–4 toxicity (OR, 1.160; 95% CI, 1.057–1.283; *p* = 0.0015). Increasing age was also independently associated with grade 3–4 toxicity (OR per 1-year increase, 1.061; 95% CI, 1.021–1.106; *p* = 0.0022), as was treatment with sunitinib compared with pazopanib (OR, 2.629; 95% CI, 1.104–6.785; *p* = 0.028). Sex was not independently associated with grade 3–4 toxicity (OR, 1.113; 95% CI, 0.469–2.574; *p* = 0.805). The full results of the Firth penalized regression analysis are presented in Table 4.

In a supportive Firth analysis using categorical BMI, obesity (BMI ≥ 30.0 kg/m^2^) was associated with higher odds of grade 3–4 toxicity compared with BMI < 25.0 kg/m^2^ (OR, 4.173; 95% CI, 1.533–11.982; *p* = 0.005). No significant difference was observed for BMI 25.0–29.9 kg/m^2^ compared with BMI < 25.0 kg/m^2^ (OR, 0.973; 95% CI, 0.380–2.458; *p* = 0.954).

Results of the conventional multivariable logistic regression analysis and the exploratory BSA analysis are provided in Supplementary Tables S1 and S2, respectively.

## 4. Discussion

In the present retrospective study, we evaluated whether anthropometric characteristics were associated with the tolerability of fixed-dose tyrosine kinase inhibitors (TKIs) used for the treatment of mRCC. The results indicated that BMI was significantly associated with treatment-related toxicity. Patients with higher BMI exhibited a significantly higher rate of grade ≥ 3 treatment-related. Importantly, this association remained significant in the Firth penalized multivariable analysis after adjustment for age, sex, and treatment type. Each 1 kg/m^2^ increase in BMI was associated with a 16% increase in the odds of grade 3–4 toxicity. In the supportive categorical analysis, obesity (BMI ≥ 30 kg/m^2^) was also associated with higher odds of grade 3–4 toxicity compared with BMI < 25 kg/m^2^.

Sunitinib and pazopanib are relatively lipophilic agents. Both agents exhibit considerable interindividual pharmacokinetic variability, and previous studies have demonstrated associations between systemic drug exposure and treatment-related toxicity [14,15,16]. However, whether BMI or adipose tissue directly contributes to variability in drug exposure remains uncertain.

Among patients receiving sunitinib, the significantly higher incidence of adverse events in overweight and obese individuals suggested that body composition-related differences may contribute to treatment tolerability. Previous studies have also supported an association between body composition and sunitinib-related toxicity. Park et al. reported that CT-defined visceral adiposity was independently associated with sunitinib-induced dose-limiting toxicity in patients with metastatic clear-cell RCC [12].

Similarly, among patients treated with pazopanib, the significantly higher incidence of grade 3–4 toxicities observed in the obese group were noteworthy. Previous pharmacokinetic studies have demonstrated substantial interindividual variability in pazopanib exposure and have reported associations between higher pazopanib trough concentrations and treatment-related toxicity [16]. However, whether body composition contributes to this variability remains unclear. Although sunitinib and pazopanib have comparable antitumor efficacy, their safety and tolerability profiles differ. In the COMPARZ trial, pazopanib demonstrated noninferior efficacy compared with sunitinib and was associated with more favorable health-related quality-of-life and safety outcomes [8]. Similarly, the PISCES trial demonstrated a significant patient preference for pazopanib over sunitinib, with differences in tolerability and quality of life contributing to treatment preference [17]. These differences should be considered when interpreting the treatment-specific toxicity patterns observed in the present study.

One potential mechanism underlying these findings involves alterations in the pharmacokinetic and pharmacodynamic properties of drugs in obese individuals. Increased adipose tissue can alter the volume of distribution of some lipophilic drugs; however, the magnitude and direction of this effect are drug-specific. Furthermore, changes in liver and kidney function associated with obesity can affect drug metabolism and elimination [18].

It has also been reported that body composition may be associated with TKI-related toxicity. Huillard et al. demonstrated that patients with mRCC who had a BMI < 25 kg/m^2^ and sarcopenia experienced a higher incidence of early dose limiting toxicity during sunitinib therapy [13]. In contrast, Park et al. reported that CT-defined visceral adiposity was independently associated with early sunitinib-related dose-limiting toxicity [12]. Although these findings may appear divergent, they emphasize that BMI, skeletal muscle mass, and visceral adiposity represent distinct aspects of body composition and should not be considered interchangeable. Both studies focused on dose-limiting toxicity occurring during the first treatment cycle, whereas the present study evaluated grade 3–4 toxicity occurring throughout treatment. Overall, studies evaluating the association between fixed-dose TKI therapy and body composition remain limited.

The current findings suggest that patients with mRCC and increased BMI may warrant more careful clinical assessment of potential risks before therapy initiation and closer monitoring for adverse events during therapy.

The strengths of the present study include its real-world design, and the comparative analysis of the toxicity profiles of two widely used TKIs. Future prospective studies incorporating detailed body composition assessment and pharmacokinetic measurements could provide further insights into this field.

However, several limitations should be acknowledged. First, the retrospective study design could have resulted in missing data, thus affecting the analysis. In addition, specific adverse-event types were not systematically captured as separate variables in the study database; therefore, associations between BMI and individual toxicity phenotypes could not be evaluated. Second, BMI may not accurately reflect detailed body composition and may overlook important parameters such as muscle mass and visceral fat. The lack of imaging-based analyses could prevent the assessment of clinically relevant subgroups such as patients with sarcopenic obesity. Furthermore, as this was a single-center study, the generalizability of findings could be limited. The sample size in the pazopanib subgroup (n = 46) was relatively small, including only 11 obese patients and nine patients with grade 3–4 treatment-related toxicity. Consequently, formal interaction analysis between treatment type and BMI could not be performed, and comparisons of BMI-related toxicity between sunitinib and pazopanib should be interpreted with caution. Additionally, concomitant medication use, particularly CYP3A4 inhibitors and inducers, was not systematically captured and therefore represents a potential source of uncontrolled confounding. Baseline serum albumin levels and liver function parameters, which could affect the pharmacologically active free fraction of both agents, were not recorded and could not be incorporated into the multivariable analysis. Similarly, baseline comorbidities, including obesity-related hypertension and cardiovascular disease, which are established risk factors for TKI-related adverse events, were not systematically documented. In addition, because a documented last-contact date was not consistently available for patients alive at the time of data cutoff, median follow-up time could not be reliably estimated for the overall cohort, and this should be considered when interpreting the reported treatment duration. Complete treatment-duration data were available for 97 patients (71.3%), and differences in treatment exposure may have therefore influenced the probability of observing treatment-related toxicity. Finally, precise data regarding the timing of the first treatment-related toxicity and time-to-dose-reduction were not available, precluding a formal time-to-toxicity analysis. Future studies should systematically collect these variables and incorporate pharmacokinetic monitoring and imaging-based body composition assessments.

## 5. Conclusions

The current study suggests that a higher BMI is associated with a greater likelihood of grade 3–4 TKI-related toxicity in patients with mRCC, highlighting the potential importance of closer toxicity monitoring in patients with higher BMI. Future multicenter, prospective studies incorporating detailed body composition assessments and pharmacokinetic measurements will be essential to validate and expand these findings.

## Figures and Tables

**Table 1:** Sociodemographic and clinical characteristics of the patients (n = 136).

Variable	N	%
Age		
Mean ± SD	58.92 ± 11.20	-
Median (min-max)	59.0 (28.00–92.00)	-
BMI		
Mean ± SD	26.28 ± 4.50	-
Median (min-max)	26.43 (14.61–39.30)	-
Gender		
Male	94	69.1
Female	42	30.9
Age group		
≤65 years	94	69.1
>65 years	42	30.9
Nephrectomy		
Present	114	83.8
Absent	22	16.2
Histology		
Clear cell	108	79.4
Non-clear cell	28	20.6
IMDC risk score		
Good	16	11.8
Intermediate	73	53.7
Poor	47	34.6
ECOG performance status		
0	44	32.4
1	87	64.0
2	5	3.7
Grade 1–2 toxicity		
Present	117	86.0
Absent	19	14.0
Grade 3–4 toxicity		
Present	41	30.1
Absent	95	69.9
Treatment discontinuation		
Present	12	8.8
Absent	124	91.2
First-line treatment		
Sunitinib	90	66.2
Pazopanib	46	33.8
Mortality		
Deceased	90	66.2
Alive	46	33.8

BMI, body mass index; IMDC, International Metastatic Renal Cell Carcinoma Database Consortium; ECOG, Eastern Cooperative Oncology Group.

**Table 2:** Comparison of BMI values according to variables in patients treated with sunitinib and pazopanib.

Variable	Sunitinib n	Sunitinib BMI (Mean ± SD)	Pazopanib n	Pazopanib BMI (Mean ± SD)
Grade 1–2 toxicity				
Present	80	26.78 ± 4.24	37	26.90 ± 4.71
Absent	10	21.74 ± 2.40	9	24.26 ± 4.88
*p*		**<0.001**		0.141
Grade 3–4 toxicity				
Present	32	27.86 ± 4.51	9	30.27 ± 5.17
Absent	58	25.32 ± 4.05	37	25.44 ± 4.27
*p*		**0.008**		**0.005**
Treatment discontinuation				
Present	11	25.79 ± 3.61	1	-
Absent	79	26.28 ± 4.48	45	26.22 ± 4.74
*p*		0.488		-

BMI, body mass index; BSA, body surface area. Bold *p*-values indicate statistical significance (*p* < 0.05), from independent-samples *t*-test.

**Table 3:** Comparison of adverse events according to BMI category in the total cohort and by treatment group.

Variable	Total			Sunitinib			Pazopanib		
	BMI < 25.0 kg/m^2^N (%)	BMI 25.0–29.9 kg/m^2^N (%)	BMI ≥ 30.0 kg/m^2^N (%)	BMI < 25.0 kg/m^2^N (%)	BMI 25.0–29.9 kg/m^2^N (%)	BMI ≥ 30.0 kg/m^2^N (%)	BMI < 25.0 kg/m^2^N (%)	BMI 25.0–29.9 kg/m^2^N (%)	BMI ≥ 30.0 kg/m^2^N (%)
Grade 1–2 toxicity									
Present	45 (76.3)	45 (91.8)	27 (96.4)	30 (75)	33 (100)	17 (100)	15 (78.9)	12 (75)	10 (90.9)
Absent	14 (23.7)	4 (8.2)	1 (3.6)	10 (25)	0 (0)	0 (0)	4 (21.1)	4 (25)	1 (9.1)
*p*	0.014 ᵃ			0.001 ᵇ			0.722 ᵇ		
Grade 3–4 toxicity									
Present	14 (23.7)	11 (22.4)	16 (57.1)	11 (27.5)	11 (33.3)	10 (58.8)	3 (15.8)	0 (0)	6 (54.5)
Absent	45 (76.3)	38 (77.6)	12 (42.9)	29 (72.5)	22 (66.7)	7 (41.2)	16 (84.2)	16 (100)	5 (45.5)
*p*	0.002 ᵃ			0.074 ᵃ			0.001 ᵇ		
Treatment discontinuation									
Present	5 (8.5)	5 (10.2)	2 (7.1)	5 (12.5)	5 (15.2)	1 (5.9)	0 (0)	0 (0)	1 (9.1)
Absent	54 (91.5)	44 (89.8)	26 (92.9)	35 (87.5)	28 (84.8)	16 (94.1)	19 (100)	16 (100)	10 (90.9)
*p*	1.000 ᵇ			0.702 ᵇ			0.240 ᵇ		

ᵃ Pearson’s chi-square test; ᵇ Fisher’s exact test. *p* < 0.05 was considered statistically significant.

**Table 4:** Firth penalized multivariable logistic regression analysis for grade 3–4 treatment-related toxicity.

Variable	Adjusted OR	95% CI	*p*-Value
BMI (per 1 kg/m^2^ increase)	1.160 *	1.057–1.283	0.0015 *
Age (per 1-year increase)	1.061 *	1.021–1.106	0.0022 *
Sex (female vs. male)	1.113	0.469–2.574	0.805
Treatment (sunitinib vs. pazopanib)	2.629 *	1.104–6.785	0.028 *

CI, confidence interval; OR, odds ratio. The outcome was grade 3–4 treatment-related toxicity. BMI was modeled continuously; the model was adjusted for continuous age, sex, and treatment type. Firth penalized logistic regression was used to reduce small-sample bias. * Statistically significant (*p* < 0.05).

## Data Availability

The data presented in this study are available on request from the corresponding author due to institutional and ethical restrictions on patient privacy.

## Supplementary Materials

The following supporting information can be downloaded at https://www.mdpi.com/article/10.3390/cancers18172730/s1: Table S1: Conventional multivariable binary logistic regression analysis of factors associated with grade 3–4 treatment related toxicity; Table S2: Exploratory analysis of body surface area (BSA) according to treatment-related toxicity and treatment discontinuation.

## Abbreviations

The following abbreviations are used in this manuscript:

BMI	Body mass index
mRCC	Metastatic renal cell carcinoma
ccRCC	Clear cell renal cell carcinoma
VEGF	Vascular endothelial growth factor
TKI	Tyrosine kinase inhibitor
PFS	Progression-free survival
TTD	Time to treatment discontinuation
IMDC	International Metastatic Renal Cell Carcinoma Database Consortium
ECOG	Eastern Cooperative Oncology Group
CTCAE	Common Terminology Criteria for Adverse Events
OR	Odds ratio
CI	Confidence interval
