# Supplementary material for: Association Between Body Mass Index and Treatment Toxicity in Patients with Metastatic Renal Cell Carcinoma Treated with Sunitinib or Pazopanib
Source: Cancers (Basel). 2026 Aug 23;18(17):2730. doi: 10.3390/cancers18172730 (PMC13565252; doi:10.3390/cancers18172730)
Supplement: Supplementary file 1 [file cancers-18-02730-s001.zip › cancers-4498140-supplementary.pdf]

Supplementary Table S1. Conventional multivariable binary logistic regression analysis of factors associated with grade 3-4 treatment related toxicity

Variable	OR	95% CI	p-value
BMI (per 1 kg/m² increase)	1.174*	1.058-1.304	0.003*
Sex (male vs. female)	0.896	0.365-2.194	0.809
Age (>65 vs. ≤65 years)	3.251*	1.300-8.128	0.012*
Nephrectomy (yes vs. no)	0.578	0.169-1.975	0.382
Histology (clear cell vs. NCC)	2.255	0.617-8.249	0.219
IMDC risk (good vs. intermediate)	1.332	0.326-5.438	0.690
IMDC risk (poor vs. intermediate)	1.011	0.399-2.565	0.981
ECOG PS (0 vs. 2)	1.150	0.089-14.835	0.915
ECOG PS (1 vs. 2)	0.843	0.076-9.396	0.889
Treatment (sunitinib vs. pazopanib)	3.080*	1.082-8.770	0.035*

CI, confidence interval; ECOG PS, Eastern Cooperative Oncology Group performance status; IMDC, International Metastatic Renal Cell Carcinoma Database Consortium; NCC, non-clear cell; OR, odds ratio. *Statistically significant (P<0.05).

Supplementary Table S2. Exploratory analysis of body surface area (BSA) according to treatment-related toxicity and treatment discontinuation

Variable	Sunitinib n	Sunitinib BSA (mean±SD)	Pazopanib n	Pazopanib BSA (mean±SD)
Grade 1-2 toxicity				
Present	80	1.863±0.175	37	1.841±0.180
Absent	10	1.796±0.149	9	1.839±0.140
p		0.250		0.976
Grade 3-4 toxicity				
Present	32	1.902±0.179	9	1.822±0.233
Absent	58	1.830±0.165	37	1.845±0.156
p		0.056		0.726
Treatment discontinuation				
Present	11	1.887±0.156	1	2.080
Absent	79	1.851±0.175	45	1.835±0.169
p		0.517		-

BSA, body surface area. Bold p-values indicate statistical significance (P<0.05), from independent-samples t-test.
